# *In Vivo* Imaging of Activated Estrogen Receptors *in Utero* by Estrogens and Bisphenol A

**DOI:** 10.1289/ehp.7155

**Published:** 2004-07-21

**Authors:** Josephine G. Lemmen, Roel J. Arends, Paul T. van der Saag, Bart van der Burg

**Affiliations:** ^1^Hubrecht Laboratory, Netherlands Institute for Developmental Biology, Uppsalalaan, Utrecht, the Netherlands; ^2^Department of Pharmacology, NV Organon, Oss, The Netherlands

**Keywords:** bisphenol A, estrogen receptor, *in utero*, *in vivo*, reporter mice

## Abstract

Environmental estrogens are of particular concern when exposure occurs during embryonic development. Although there are good models to study estrogenic activity of chemicals in adult animals, developmental exposure is much more difficult to test. The weak estrogenic activity of the environmental estrogen bisphenol A (BPA) in embryos is controversial. We have recently generated transgenic mice that carry a reporter construct with estrogen-responsive elements coupled to luciferase. We show that, using this *in vivo* model in combination with the IVIS imaging system, activation of estrogen receptors (ERs) by maternally applied BPA and other estrogens can be detected in living embryos *in utero*. Eight hours after exposure to 1 mg/kg BPA, ER transactivation could be significantly induced in the embryos. This was more potent than would be estimated from *in vitro* assays, although its intrinsic activity is still lower than that of diethylstilbestrol and 17β-estradiol dipropionate. On the basis of these results, we conclude that the estrogenic potency of BPA estimated using *in vitro* assays might underestimate its estrogenic potential in embryos.

There is concern about compounds in the environment that could partially mimic the effects of estrogen, which could possibly explain the rising incidence of reproductive abnormalities and certain cancers (Miller and Sharpe 1998). These environmental estrogens are a structurally very diverse group of compounds that can only be identified as environmental estrogens by carrying out functional studies. One compound of concern is bisphenol A (BPA), a monomer component of polycarbonate plastics and epoxy resins. Humans are exposed to BPA when it leaks from plastic packaging and dental appliances (Feldman 1997), and nanomolar concentrations have been measured in human serum (Takeuchi and Tsutsumi 2002). Recently, it was reported that BPA could cause meiotic aneuploidy when female mice were exposed unintentionally through damaged cage material (Hunt et al. 2003). BPA has been found to possess weak estrogenic properties in *in vitro* assays with an EC_50_ (median effective concentration) about 10,000 times less than strong estrogens such as 17β-estradiol (E_2_) and diethylstilbestrol (DES) (Kim et al. 2001; Kuiper et al. 1998; Perez et al. 1998). However, the *in vivo* estrogenic potential of BPA can vary depending on animal species or strain studied (Milligan et al. 1998; Steinmetz et al. 1997, 1998). In addition, the end point is very important. It was shown that BPA did induce DNA synthesis in vaginal epithelium of Fischer 334 rats but did not in Sprague-Dawley rats, whereas in both strains BPA increased c-*fos* mRNA expression (Long et al. 2000). Two classical *in vivo* assays, the rodent uterine wet weight assay and the vaginal cornification assay, have traditionally been used for testing estrogenic activity of compounds. In these assays, BPA has been found to be active (Ashby and Tinwell 1998; Markey et al. 2001; Papaconstantinou et al. 2000) as well as inactive (Coldham et al. 1997; Gould et al. 1998; Mehmood et al 2000; Tinwell et al. 2000). When found active, its potency was four orders of magnitude lower than that of DES, confirming the weak estrogenicity measured in *in vitro* assays.

It has been proposed that the developing embryo may be much more susceptible to harmful effects of environmental estrogens compared with adult animals (Bigsby et al. 1999; Dencker and Eriksson 1998; McLachlan 2001; Miller 1983). The best-known example of a developmentally active compound is the synthetic estrogen DES, which was prescribed from the 1940s until the 1970s to prevent miscarriages. Children exposed to DES *in utero* developed abnormalities and cancer of the reproductive tract, whereas these effects were not found in their mothers (Herbst et al. 1971; McLachlan et al. 1975). Structural similarities between DES and BPA are evident, and it has been suggested that prenatal exposure to BPA may cause abnormalities similar to those elicited by DES (vom Saal et al. 1998). Experiments examining the estrogenic effects of BPA on embryos have led to contradictory findings. Although some studies have reported prostate enlargement in offspring of BPA-exposed mice (Howdeshell et al. 1999), others reported no effect (Ashby et al. 1999; Cagen et al. 1999; Nagao et al. 2002; Welshons et al. 1999). Levels of BPA in amniotic fluid at 15–18 weeks of gestation have been shown to be 5-fold higher than serum levels in both pregnant and nonpregnant women, suggesting a possible accumulation of BPA in the early embryo (Ikezuki et al. 2002), although Domoradzki et al. (2003) could not confirm this in animal experiments. Unfortunately, there is no model in which estrogen effects can be determined directly in embryos.

We have developed an approach, using transgenic reporter mice, that allows us to determine direct activation of estrogen receptor (ER) signaling in embryos. For this, we used our recently established transgenic mice model (Lemmen et al. 2004); direct activation of ERs is detected photometrically by measuring luciferase activity, allowing both quantitative and time-course analysis of estrogen target gene activation *in vivo*. Other estrogen reporter mice that have been generated do not exclude estrogen-response-element (ERE)–independent activation because of the presence of other promoter sequences (Ciana et al. 2001; Nagel et al. 2001; Toda et al. 2004). In our model, activation of the construct via promoter sites other than the EREs is avoided by using only a minimal TATA box in the construct, resulting in low background activity. Although in natural promoters ERE sequences are often found together with other enhancer sequences and other ways of ER transactivation (e.g., via AP1 sites) are possible, we chose a reductionistic approach, with only ERE sequences in the synthetic promoter used. In the present study, we used this model to examine the ability of BPA—in comparison with known strong estrogens DES and 17β-estradiol dipropionate (EP)—to activate endogenous ERs present in mouse embryos (Lemmen et al. 1999). Surprisingly, we found BPA to be more potent in activating embryonic ERs than would be expected on the basis of its *in vitro* activity.

## Materials and Methods

### Transgenic animals.

We used transgenic animals carrying a reporter construct that consists of three estrogen-responsive elements (GAGCTTAGGTCACTGTGACCT) upstream of a minimal human E1B TATA promoter sequence (GGGTATATAAT) coupled to luciferase surrounded by chicken β-globin insulator (Chung et al. 1993) sequences (Lemmen et al. 2004). To obtain transgenic embryos, heterozygote transgenic males from line INS3 were mated with wild-type females (F_1_ from C57Bl/6J × CBA). Heterozygote males were used so that every litter would also contain wild type embryos, which could serve as an internal negative control. Females were checked daily for the presence of a vaginal plug, and when a plug was detected, that day was designated 0.5 day postcoitum (dpc).

### Compounds and exposures.

E_2_, EP, DES, and BPA were all purchased from Sigma-Aldrich (Roosendaal, The Netherlands). For injections, compounds were dissolved in corn oil (Sigma-Aldrich) at a concentration of 10 mg/mL and then diluted in 1:10 steps in corn oil to the required doses (DES, 10–1,000 μg/kg; EP, 10–10,000 μg/kg; BPA, 10–10,000 μg/kg). Compounds or vehicle were injected intraperitoneally in 13.5 dpc pregnant animals. Animal experiments were performed with approval of the Netherlands Academy of Arts and Sciences Animal Ethics Committee. The IVIS imaging experiments were done with additional approval of the Animal Ethics Committee of NV Organon.

### In vivo *luciferase measurement.*

With the Xenogen IVIS imaging system (Xenogen, Alameda, CA, USA), luciferase activity was monitored in living animals 0, 2, 4, 8, and 24 hr after compound injection. Animals were injected subcutaneously with luciferin (150 μL, 30 mg/mL). After 15 min, the animals were placed in a dark imaging chamber under isoflurane anesthesia. Resulting photon emission from the luciferin/luciferase reaction was detected with a CCD (charge-coupled device) camera. The photon image obtained was superimposed on a normal video image of the mouse with Living Image software (Xenogen). We used IGOR software (WaveMetrics Corp., Lake Oswego, OR, USA) to quantify the photon signal over the area encompassing the embryos. For all pregnant animals, this area was kept of equal size.

### Luciferase measurement lysates.

Embryos were isolated either at 8 or 24 hr after compound injection and frozen at –80°C. When embryos were isolated from the amniotic membranes, we kept tails separated and stored at –20°C for subsequent DNA isolation and polymerase chain reaction for presence of the transgene, as described previously (Legler et al. 2000). Only transgenic embryos were used for further luciferase measurement. Subsequently, embryos were thawed on ice and lysis buffer [1% (vol/vol) Triton X-100, 2.5 × 10^−2^ M glycylglycine, 1.5 × 10^−2^ M MgSO_4_, 4 × 10^−3^ M EGTA, and 1 × 10^−3^ M dithiothreitol (DTT)] was added. Next, samples were sonicated, the lysate centrifuged, and the supernatant collected. Samples (25 μL in duplicate) were analyzed for luciferase enzyme activity in a luminometer (LUMAC/3M BV, Schaesberg, The Netherlands) with injection of 100 μL luciferin substrate as previously described (Lemmen et al. 2004). Luciferase activity was corrected for protein content as measured with the Bradford assay.

### In vitro *estrogenic activity assay in stable cell lines.*

Stable 239HEK cell lines containing human ER-α(hER-α) or hER-βand an estrogen-responsive reporter construct—similar to the one introduced in the transgenic animals, but without the flanking insulator sequences—were used and cultured as previously described (Lemmen et al. 2002). Briefly, cells were plated in 96-well tissue culture plates (NUNC, Life Technologies, Breda, The Netherlands) in medium consisting of phenol red–free Dulbecco’s modified Eagle’s medium/F12 (1:1) medium containing 3 × 10^−8^ M selenite, 10 μg/mL transferrin, 0.2% (wt/vol) bovine serum albumin, and 5% (wt/vol) dextran-coated charcoal-stripped fetal calf serum. After 24 hr, medium was refreshed and after another 24 hr, the medium was removed and fresh medium containing test compounds (dissolved in ethanol) was added. After 24 hr of incubation, the medium was removed and 50 μL lysis solution [1% (vol/vol) Triton X-100, 2.5 × 10^−2^ M glycylglycine, 1.5 × 10^−2^ M magnesium sulfate, 4 × 10^−3^ M EGTA, and 1 × 10^−3^ M DTT] was added directly to the cells. Luciferase activity of 25 μL cell lysate was measured with the Luclite Luciferase Reporter gene assay kit (Perkin-Elmer, Brussels, Belgium) according to the manufacturer’s instructions using 25 μL Luclite solution on a Topcount liquid scintillation counter (Perkin-Elmer).

### Data analysis and statistics.

We considered one litter as a statistical unit rather than one embryo because we assumed that all embryos in one litter were subject to the same variation of the compound injection and placental transfer. Therefore, the average ± SEM per litter was calculated, and these were then averaged to express the luciferase activity per group. All data were log-transformed and tested for normality with the Shapiro-Wilks test using SPSS 12.0 (SPSS, Chicago, IL, USA). The data were not normally distributed; therefore, we determined significant differences of treatment groups from oil-exposed control using Kruskal-Wallis analysis followed by the Dunn’s posttest using GraphPad Prism 3.02 (GraphPad Software Inc., San Diego, CA, USA). In addition, the presence of a linear trend in the dose response was determined by analysis of variance followed by a posttest for linear trends using GraphPad Prism 3.02. For the IVIS time-course experiments, we performed a Friedman test followed by Dunn’s posttest using GraphPad Prism 3.02.

The *in vitro* dose–response activation curves obtained with the stable cell lines were fitted using the sigmoidal fit {*y* = *a*_0_ + *a*_1_/1 + exp[–(*x* – *a*_2_)/*a*_3_]} in Slidewrite Plus for Windows (version 3.0; BIS, Ridderkerk, The Netherlands), which determines the fitting coefficients by an iterative process minimizing the c2 merit function (least squares criterion). The EC_50_ (median effective concentration) values were calculated by determining the concentration by which 50% of maximum activity was reached using the sigmoidal fit equation. The cell line data shown are the average of at least two independent experiments with each experimental point performed in triplicate. Data are shown as a percentage of maximal induction by E_2_.

## Results

### Estrogens activate endogenous ERs in transgenic embryos.

To be able to measure luciferase activity in the transgenic embryos with the IVIS system, it was crucial that wild-type mothers carry the transgenic embryos. If transgenic mothers had been used, strong photon emission would have been generated after the estrogen and luciferin injections, masking the signal emitted from embryos. We chose 13.5–14.5 dpc as the time for exposure because at this time point ERs are expressed in the embryo (Lemmen et al. 1999) and because this is a sensitive time point for disruption of reproductive organs by prenatal estrogen exposure. In nonexposed embryos, we detected no luciferase activity with IVIS and barely any luciferase activity in embryo lysates.

**Figure 1:**
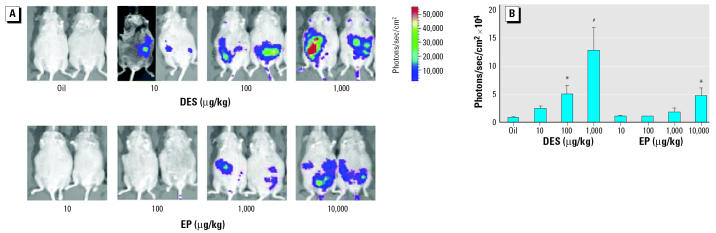
*In vivo* activation of estrogen-responsive reporter construct by DES and EP in embryos. (A) In vivo activation of estrogen-responsive reporter construct (luciferase) by DES and EP in 13.5 dpc transgenic embryos measured with IVIS; the number of photons produced by the reaction between luciferase and luciferin is depicted in a color image superimposed on a video image of the pregnant animal. (*B*) Quantification of the signal produced in the embryos after DES and EP exposure. Values shown are mean ± SEM for oil (*n* = 5 litters), DES 10 μg/kg (*n* = 4), DES 100 μg/kg (*n* = 4), DES 1,000 μg/kg (*n* = 6), EP 10 μg/kg (*n* = 4), EP 100 μg/kg (*n* = 2), EP 1,000 μg/kg (*n* = 5), and EP 10,000 μg/kg (*n* = 6). Abscissa, dose of DES/EP or oil; ordinate, photons/sec/cm^2^ measured in an area of the pregnant mouse encompassing the embryos.
*p < 0.05,
^#^*p* < 0.001 compared with oil-exposed mean as determined by Kruskal-Wallis analysis followed by the Dunn’s posttest.

**Figure 2:**
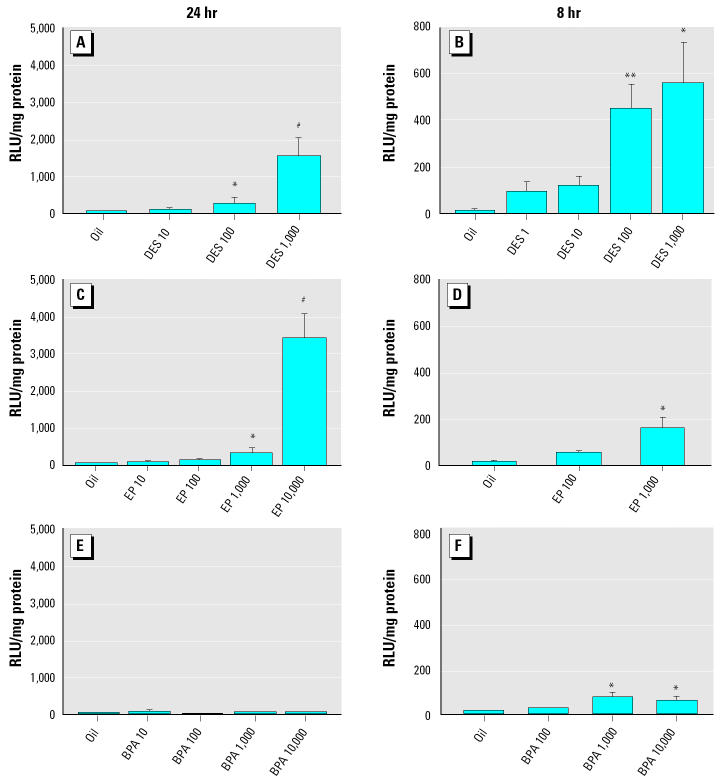
*Ex vivo* measurement of embryo lysates of *in utero* activated estrogen-responsive reporter construct (luciferase) 8 and 24 hr after exposure to DES (*A, B*), EP (*C, D*), and BPA (*E, F*). Values shown are mean ± SEM for the 24 hr measurements: oil (*n* = 9 litters) DES 10–1,000 μg/kg (*n* = 8–9), EP 10–100 μg/kg (*n* = 5), EP 1,000–10,000 μg/kg (*n* = 7–8), BPA 10–100 μg/kg (*n* = 2), and BPA 1,000–10,000 μg/kg (*n* = 6–8); and for the 8 hr measurements: oil (*n* = 3), DES 1 μg/kg (*n* = 2), DES 10 μg/kg (*n* = 5), DES 100 μg/kg (*n* = 8), DES 1,000 μg/kg (*n* = 3), EP 100 μg/kg (*n* = 3), EP 1,000 μg/kg (*n* = 5), BPA 100 μg/kg (*n* = 2), BPA 1,000 μg/kg (*n* = 8), and BPA 10,000 μg/kg (*n* = 5). Abscissa, dose of DES, EP, BPA, or oil; ordinate, Luc-units/mg protein as measured on luminometer (LUMAC).
*p < 0.05,
**p < 0.01,
^#^*p* < 0.001 compared with oil-exposed mean as determined by Kruskal-Wallis analysis followed by the Dunn’s posttest.

*In utero* luciferase activity in transgenic embryos was induced dose dependently by DES and EP. When measured 8 hr after exposure, 100 and 1,000 μg/kg DES significantly induced luciferase activity when assessed with the IVIS system (Figure 1) and in embryo lysates *ex vivo* measured in the luminometer (Figure 2). No plateau levels in luciferase activity were reached, and the profile of induction after DES exposure was similar for both methods used to assess luciferase activity. For EP only, the 10,000 μg/kg dose was able to significantly induce luciferase activity when measured with IVIS after 8 hr (Figure 1). When measured *ex vivo* in embryo lysates, 1,000 μg/kg EP already significantly induced luciferase activity (Figure 2). Fold induction of luciferase activity of estrogen exposed over controls 8 hr after exposure was, however, lower using IVIS compared with measurements in lysates. For DES doses of 100 and 1,000 μg/kg, induction was 5-fold and 14-fold greater, respectively, when measured with the IVIS system, whereas it was 41-fold and 51-fold greater, respectively, when measured *ex vivo* on embryo lysates. However, these differences in induction are likely based on a difference in noise rather than in signal. Also, for EP inductions were larger when measured *ex vivo* than when measured with IVIS (data not shown).

**Figure 3:**
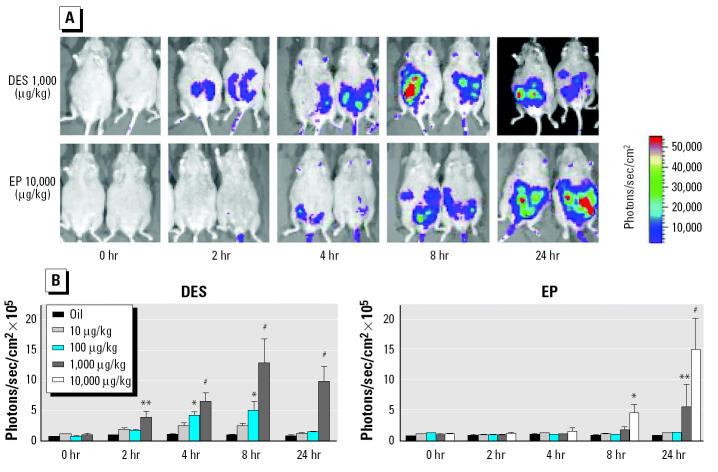
*In utero* time course of activation of estrogen-responsive reporter construct by DES and EP in embryos. (*A*) *In utero* time course of activation of estrogen-responsive reporter construct (luciferase) by DES and EP in transgenic embryos measured with IVIS. The number of photons is depicted in a color image superimposed on a video image of the pregnant animal. (*B*) Quantification of the signal produced in embryos after DES and EP exposure. Values shown are mean ± SEM for oil (*n* = 5 litters), DES 10 μg/kg (*n* = 4), DES 100 μg/kg (*n* = 4), DES 1,000 μg/kg (*n* = 6), EP 10 μg/kg (*n* = 4), EP 100 μg/kg (*n* = 2), EP 1,000 μg/kg (*n* = 5), and EP 10,000 μg/kg (*n* = 6). The 10,000 μg/kg dose was not tested for DES. Abscissa, time in hours after hormone exposure; ordinate, photons/sec/cm^2^ measured in an area of the pregnant mouse encompassing the embryos.
*p < 0.05,
**p < 0.01,
^#^*p* < 0.001 compared with oil-exposed mean as determined by Kruskal-Wallis analysis followed by the Dunn’s posttest.

An important advantage of using the IVIS system is that the luciferase induction can be followed in time in a single animal, making it a very useful tool for obtaining information on the kinetics of tissue distribution and gene activation by compounds. With the IVIS measurements, we observed a difference between DES and EP in the kinetics of inducing luciferase activity (Figure 3). DES (1,000 μg/kg) significantly induced luciferase activity, exceeding levels in oil-exposed animals 2 hr after exposure, and this activity peaked 8 hr after exposure (Figure 3). In contrast, only 8 hr after EP exposure (10,000 μg/kg), luciferase activity was significantly above levels in oil-exposed animals, with a peak at 24 hr after exposure (Figure 3). This difference in kinetics thus complicates comparing relative potencies of these estrogens to induce luciferase activity. At 24 hr after estrogen exposure, the embryos were isolated and luciferase activity was measured *ex vivo*, showing that 100 and 1,000 μg/kg DES and 1,000 and 10,000 μg/kg EP were able to significantly induce luciferase activity compared with oil-exposed controls (Figure 2).

### BPA activates ERs in transgenic embryos.

**Figure 4:**
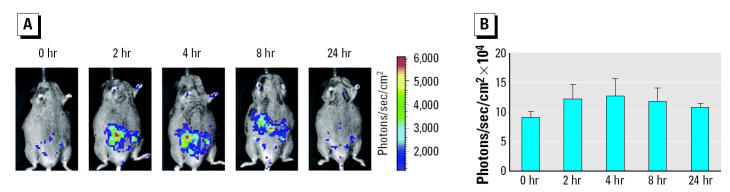
*In utero* time course of activation of estrogen-responsive reporter construct by BPA in embryos. (*A*) *In utero* time course of activation of estrogen-responsive reporter construct (luciferase) by 10,000 μg/kg BPA in transgenic embryos measured with IVIS. The number of photons is depicted in a color image superimposed on a video image of the pregnant animal. (*B*) Quantification of the signal produced in embryos after BPA exposure. Values shown are mean ± SEM (*n* = 5). Abscissa, time in hours after hormone exposure; ordinate, photons/sec/cm^2^ measured in an area of the pregnant mouse encompassing the embryos. Note that the scale differs from those in Figures 1 and 3.

Eight hours after exposure to 10,000 μg/kg BPA, luciferase activity was higher than in oil-exposed animals when measured with IVIS (Figure 4), although this was not statistically significant. To be able to visualize the weak BPA signal, the scale bar of the superimposed video image had to be adjusted compared with Figures 1 and 3. When lysates from embryos sacrificed 24 hr after exposure were measured, no difference between oil- and BPA-exposed animals was found (Figure 2). Because BPA, like DES, may enter the fetal circulation rapidly (Miyakoda et al. 1999; Takahashi and Oishi 2000), other embryos were isolated 8 hr after exposure to 100 and 1,000 μg/kg BPA, EP, and DES or oil. At this time point, luciferase activity was significantly higher after exposure to 1,000 and 10,000 μg/kg BPA compared with oil-exposed animals (Figure 2). Therefore, at least at early time points, BPA is able to transactivate the embryonic ERs and resembles DES rather than EP in its kinetics of luciferase activation.

### In vitro *potency of estrogens and BPA.*

**Figure 5:**
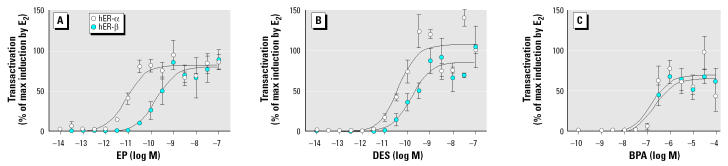
Transactivation of hER-α and hER-βby DES (*A*), EP (*B*), and BPA (*C*) presented as a percentage of maximal induction by E_2_. Values shown are mean ± SEM of three independent experiments done in triplicate. Abscissa, log molar concentration of hormone; ordinate, transcriptional activity as percentage of maximal induction by E_2_ for each ER subtype.

The compounds used for the exposure experiments of transgenic animals were also tested in an *in vitro* assay to separately assess their potency to activate ER-αor ER-βusing a similar reporter gene as used in the transgenic animals, only without the flanking insulator sequences (Figure 5). All three compounds activated hER-αand hER-β*in vitro*. The EC_50_ values for ER-αwere 3.9 × 10^−11^ M, 8.5 × 10^−12^ M, and 1.6 × 10^−7^ M for DES, EP, and BPA, respectively. EC_50_ values for ER-βwere 3.9 × 10^−11^ M, 8.5 × 10^−12^ M, and 1.6 × 10^−7^ M for DES, EP, and BPA, respectively. In these experiments, BPA was found to be 5,000 times less active than DES in activating ER-αand 1,400 times less active than DES toward ER-β transactivation. EP was 4.6 times more potent than DES in activating hER-αand just as potent as DES in activating hER-β. These results confirm the reported weak estrogenicity of BPA *in vitro*.

## Discussion

We successfully applied our new sensitive estrogen reporter mice to assess the ability of DES, EP, and BPA to activate ER signaling in embryos. In the present study, BPA exposure of pregnant mice induced the estrogen reporter through activation of endogenous ERs in mouse embryos. Hence, the generated *in vivo* model was successful in detecting estrogenic activity of a suspected environmental estrogen in embryos exposed *in utero*. In addition, our results show that *in utero* activation of ERs by BPA, at early time points after exposure, requires much lower doses than extrapolations from *in vitro* measurements would predict.

Because barely any luciferase activity could be measured in nonexposed embryos, we concluded that either there are no active endogenous estrogens during the life stage tested (13–14.5 dpc), or that our model is not sufficiently sensitive to detect their presence. Very low levels of estrogens have been described to be present in steroid extracts of mouse embryo homogenates as determined in estrogenic activity measurements (Lemmen et al. 2002). These levels may, however, be too low to activate endogenous ERs or are not able to activate ERs *in vivo* because of such different factors as tissue distribution and inactivation through binding proteins. It is possible that measurements of luciferase activity on dissected organs from embryos would prevent dilution of the luciferase signal below the detection limit. The low sensitivity of our model is also apparent in the high doses of DES and EP needed to be able to show a significant luciferase induction (Figures 1 and 2).

From measurements taken after mice pregnant with transgenic embryos were exposed to DES and EP, it was possible to evaluate the ability of the *in vivo* model to detect well-known estrogens in embryos exposed *in utero*. Exposure to DES showed a dose- and time-dependent induction of luciferase activity. The kinetic data obtained with the IVIS system showed that for all DES doses peak activity occurred at 8 hr after exposure. Previous studies using ^14^C-DES have shown that upon injection of pregnant mice, fetal plasma levels reach a peak after 2 hr and then disappear slowly (McLachlan 1977). The time difference in induction of maximal luciferase activity (i.e., after 8 hr) compared with an expected earlier DES peak in fetal plasma (i.e., after 2 hr) may be additionally due to the time required for transcription and translation of luciferase. Comparing DES with EP, it is evident that EP also shows a dose-dependent increase in luciferase activity. However, the EP-induced peak of luciferase activity was not seen before 24 hr after exposure. The observed time course of EP-induced luciferase activation could be due to a slow transfer to the embryos of EP itself or a relatively slow uptake by target tissues. Because no data are available on the kinetics of placental transfer of EP, only data on placental transfer of E_2_ can be used for comparison. In rhesus monkeys, placental transfer of ^14^C-DES and ^14^C-E_2_ was similar (Hill et al. 1980). Similar to embryos, exposure of adult transgenic animals to EP induced peak activation of luciferase at 24 hr after exposure (Lemmen et al. 2004), suggesting that a difference in placental transfer is unlikely to explain the delay in activation of luciferase by EP compared with DES. Another explanation for the difference observed between EP and DES exposure in peak luciferase activity could be that EP is initially bound to binding proteins in the serum and uptake by the embryonal target tissues is therefore slower compared with DES, which has much lower affinity to binding proteins (Arnold et al. 1996; Simmons et al. 1994). However, when E_2_ was tested in adult animals (Lemmen et al. 2004), it did show a peak in luciferase activity at 8 hr rather than at 24 hr; because E_2_ is bound to binding proteins as is EP, this suggests that the time needed for removal of the propionate groups could explain the difference in kinetics between EP and DES.

In pregnant rats, BPA has been shown to enter the fetal circulation with a peak concentration after 15–20 min (Takahashi and Oishi 2000). When exposing pregnant mice to 100 mg/kg BPA given subcutaneously, BPA was detected 30 min after exposure in fetal sera, liver, brain uterus, and testes (Domoradzki et al. 2003; Shin et al. 2002; Uchida et al. 2002). In the present study, BPA was found to significantly induce luciferase activity at doses of 1,000 and 10,000 μg/kg 8 hr after exposure. The kinetics of luciferase induction by BPA, measured with the IVIS system, resemble the profile of DES. Although the molecular structure of BPA and DES is similar, it remains unknown whether this contributes to the similarity in their kinetics in inducing luciferase activity. Testing more estrogenic compounds with various structures could shed light on this question.

Like DES, BPA showed a transient induction of luciferase activity in embryos; thus, estrogenic potency of BPA is compared with DES rather than with EP. *In utero* luciferase activation by BPA in transgenic embryos at 8 hr after exposure was significant from oil-exposed controls with 1 mg/kg BPA. Likewise, Nagel et al. (2001) found a significant increase in ER transcriptional activity in the adult uterus after exposure to 1 mg/kg BPA, whereas this dose did not induce a uterine wet weight response. DES was significantly different from oil-exposed controls at a dose of 100 μg/kg (only 10 times less than BPA), which suggests a high *in vivo* estrogenic potency of BPA. It should be noted that doses of 1 and 10 μg/kg DES induce almost a similar transcriptional activation (Figure 2), and the activation is approximately 20% of maximal activity induced by DES. *In vitro*, BPA was three to four orders of magnitude less active than DES, consistent with previous reports (Andersen et al. 1999; Kim et al. 2001; Kuiper et al. 1998). Thus, in our hands the relative potency of BPA seems to be higher *in utero* than *in vitro* on ER-α, which is the most abundantly expressed ER during embryogenesis (Lemmen et al. 1999). We believe this difference is not due to the use of human ERs *in vitro* versus the endogenous mouse ERs *in utero*. It has been shown that human and mouse ER-αhave the same affinity for DES and BPA (Matthews et al. 2000), and this is likely to be the case for ER-βas well. One explanation for a higher estrogenic potency of BPA *in utero* versus *in vitro* could be that *in vivo* BPA is converted to metabolites with enhanced estrogenicity (Ben-Jonathan and Steinmetz 1998; Yoshihara et al. 2004), although others have shown that BPA is mainly metabolized to a less active metabolite BPA monoglucuronide (Domoradzki et al. 2003; Pottenger et al. 2000). Another explanation could be that BPA has a lower affinity for the steroid-binding proteins present in serum, giving it a higher bioavailability than EP, a factor that is not taken into account in the *in vitro* assay. However, we feel this cannot explain the *in vivo* versus *in vitro* potency difference as we compare BPA with DES, and DES does not have a high affinity for binding proteins.

Strain differences in sensitivity to estrogens have been reported. The strain used in this study, C57Bl/6J (B6), has been shown to be more sensitive than CD-1 mice with respect to reduction of testis weight after estrogen exposure (Spearow et al. 1999). Also, for other end points of estrogen exposure, the B6 strain has been shown to be a sensitive strain (Roper et al. 1999; Spearow et al. 2001). In CFLP mice, 0.5 mg/mouse (~ 16.7 mg/kg) BPA was reported to be inactive in the uterine wet weight assay, whereas the other dose tested (5 mg/mouse, ~ 167 mg/kg) was toxic (Coldham et al. 1997). In CD-1 mice, a uterotrophic response was induced by 100 mg/kg BPA (Markey et al. 2001), whereas in B6C3F1 mice, doses between 0.8 and 8 mg/kg could induce uterine wet weight increase (Papaconstantinou et al. 2000). In the present study, a significant induction of luciferase activity *in utero* was detected after administration of 1 mg/kg BPA to pregnant females. The use of nontransgenic mother animals with a pure B6 background rather than the B6/CBA cross used could further increase the sensitivity of the present model.

In conclusion, we have shown that the mouse model presented here can be used to detect activation of ERs by maternally applied BPA and that other estrogens can be detected in living embryos *in utero*. BPA was more potent than would be estimated from *in vitro* assays, although its intrinsic activity is still lower than that of DES and EP. On the other hand, effects on individual embryonic organs might be larger and could be underestimated because we measured total embryo lysates. When considering that nanomolar levels of BPA have been measured in human serum (Takeuchi and Tsutsumi 2002), human amniotic fluid at 15–18 weeks of gestation (Ikezuki et al. 2002), and surface water (Fromme et al. 2003), concern about BPA exposure during embryonic/fetal life seems to be justified. It should be noted, however, that in our model the BPA effect had a more transient nature than did that of the other hormones. If and how this will translate to a biological effect in the exposed embryos should be the target of further investigations using other approaches.
